# Initial Expectations and Confidence Affect the Formation of Novel Self-Beliefs and Their Revision

**DOI:** 10.1162/OPMI.a.36

**Published:** 2025-10-29

**Authors:** Alexander Schröder, Nora Czekalla, Annalina V. Mayer, Lei Zhang, David S. Stolz, Christoph W. Korn, Susanne Diekelmann, Finn Luebber, Frieder M. Paulus, Laura Müller-Pinzler, Sören Krach

**Affiliations:** Social Neuroscience Lab, Department of Psychiatry and Psychotherapy, University of Lübeck, Lübeck, Germany; Center of Brain, Behavior and Metabolism (CBBM), University of Lübeck, Lübeck, Germany; Centre for Human Brain Health, School of Psychology, University of Birmingham, Birmingham, UK; Institute for Mental Health, School of Psychology, University of Birmingham, Birmingham, UK; Section Social Neuroscience, Department of General Psychiatry, University of Heidelberg, Heidelberg, Germany; Institute of Medical Psychology and Behavioral Neurobiology, University of Tübingen, Tübingen, Germany

**Keywords:** belief formation, belief revision, social learning, computational modeling, confirmation bias, confidence

## Abstract

Human self-beliefs hinge on social feedback, but their formation and revision are not solely based on new information. Biases during learning, such as confirming initial expectations, can lead to inaccurate beliefs. This study uses computational modeling to explore how initial expectations about one’s own and others’ abilities and confidence in these beliefs affect processes of belief formation and belief revision in novel behavioral domains. In the first session, participants formed performance beliefs through trial-by-trial feedback. In the second session, feedback contingencies were reversed to promote a revision of beliefs. Results showed that people form and revise beliefs in a confirmatory manner, with lower initial expectations being linked to more negatively biased belief formation and revision, while growing confidence strengthened these beliefs over time. Once formed, these beliefs proved resistant to change even when faced with contradictory feedback. The findings suggest that newly formed beliefs become entrenched and resistant to new, contradictory information in a short period of time. Understanding how self-beliefs are formed, the role that confidence plays in this process, and why established beliefs are difficult to revise can inform the development of interventions aimed at promoting more adaptive learning in educational, clinical, and social contexts.

## INTRODUCTION

Continuously learning and updating beliefs about one’s personality, appearance, health or abilities is a fundamental prerequisite for successfully coping with everyday life. These beliefs are shaped in social situations where people frequently receive feedback on these aspects (Czekalla et al., 2021; Müller-Pinzler et al., 2019, 2022; Will et al., 2020). To adapt effectively and pursue future goals, integrating such feedback into existing beliefs is essential (Bandura & Locke, 2003). However, this feedback may conflict with how we previously thought. For instance, someone might believe they are funny, only to later observe that others do not respond with laughter. According to social psychological theory, such discrepancies generate cognitive dissonance, which individuals are motivated to resolve (Festinger, 1957). This can occur by revising the initial belief, adjusting the view of oneself as funny, or by dismissing or reinterpreting the feedback to preserve the original belief. This study aims to investigate the mechanisms that influence the formation and possible revision of beliefs.

Two components are critical in understanding how people form and revise their beliefs: First, belief updates depend on existing expectations against which new information is compared. In the above example, a perceived mismatch occurs only if there was already a certain expectation regarding the extent of one’s humor. Second, whether individuals update their beliefs in the face of disconfirming evidence also hinges on the confidence they place in their expectations. For example, strong confidence in one’s sense of humor may reduce the likelihood of revising this belief following disconfirming social feedback. Despite their importance, the exact role of initial expectations and confidence in determining whether beliefs regarding the performances of an agent are maintained or revised in the face of conflicting feedback remains unclear.

Regarding the potential impact of existing expectations, previous research has shown that individuals do not integrate information completely balanced to arrive at the most accurate belief possible. Instead, the process of belief formation, i.e., the way information is integrated and used to build up a belief, is inherently biased (Loewenstein, 2006; Sharot & Garrett, 2016). Recent computational modeling advances have enabled the formal description of the mechanisms underlying biases in belief formation through feedback-driven learning. Here, reinforcement learning models show that beliefs are formed and updated based on the prediction errors (PE), i.e., the mismatch between existing prior expectations and feedback (den Ouden et al., 2012; Lockwood & Klein-Flügge, 2021; Sutton & Barto, 2018; Zhang et al., 2020). There is now ample evidence proving that the valence of the PEs (i.e. positive versus negative) may differently impact how beliefs are updated following new information (Müller-Pinzler et al., 2019; Sharot & Garrett, 2016). For example, when confronted with information on desirable traits such as intelligence or beauty, people tend to update their beliefs more strongly after positive than negative prediction errors (Eil & Rao, 2011; Korn et al., 2012, 2014). This positivity bias was also found when participants were confronted with feedback on the likelihood of experiencing future adverse life events (Korn et al., 2012; Kuzmanovic & Rigoux, 2017; Mobius et al., 2011; Sharot et al., 2011, 2012), leading them to believe that they are less likely to be negatively affected. A possible explanation for this positivity bias is that such beliefs not only help maximize external outcomes through the most precise models of ourselves but also carry intrinsic value on their own (Bénabou & Tirole, 2016; Bromberg-Martin & Sharot, 2020). Taking the above example, believing that you are a funny person can lead to a positive self-evaluation, which people potentially strive to maintain. The process of how novel information is integrated may thus be biased in support of the positive self-evaluation (Bromberg-Martin & Sharot, 2020; Cogliati Dezza et al., 2022; Sharot et al., 2023).

In contrast, there are also occasions where it may be beneficial to assign greater weight to negative over positive feedback when updating beliefs. For example, individuals update self-beliefs more strongly after receiving worse-than-expected feedback in a performance context (Czekalla et al., 2021; Müller-Pinzler et al., 2019, 2022; Zamfir & Dayan, 2022), arguably because this feedback indicates potential for improvement (Müller-Pinzler et al., 2019). This negativity bias occurred specifically in situations where participants received self-related information on their performance but was not evident when participants processed similar feedback about another person (Müller-Pinzler et al., 2019, 2022). Such stronger integration of negative feedback was also discussed as helpful in achieving more coherence and consistency in self-beliefs (Mokady & Reggev, 2022). Further, several studies have shown that individuals are motivated to maintain consistency in beliefs about themselves or the world by processing information in a way that is biased by their initial beliefs. This is often referred to as ‘confirmation bias’ (Hart et al., 2009; Kaanders et al., 2022; Klayman, 1995; Palminteri, 2023; Palminteri & Lebreton, 2022; Talluri et al., 2018).

Besides maintaining consistency, another component that influences whether beliefs are maintained or revised, at least to a certain extent, is confidence, i.e., the subjective certainty about whether a belief is actually correct (Fleming, 2024). Based on probabilistic learning tasks, it was shown that confidence guides updating behavior, in which confidence controls the weight of new evidence and how much is learned from new information (Meyniel & Dehaene, 2017; Meyniel et al., 2015). Further, increasing confidence dampens electrophysiological responses to surprising information (Meyniel, 2020) and limits how available information guides perceptual decision (Balsdon et al., 2020).

Previous studies have examined confidence in the context of decision-making under uncertainty, such as choices between stimuli with volatile reward probabilities (Meyniel & Dehaene, 2017; Meyniel et al., 2015). Yet, confidence is rarely assessed in studies focusing on more complex beliefs, such as those pertaining to beliefs about one’s own or others’ abilities or personality traits. Nevertheless, it is reasonable to assume that individuals’ confidence in their beliefs increases once they have been established through repeated feedback. Consequently, beliefs that have consistently proven predictive may be more resistant to change than beliefs about topics that are unfamiliar or weakly held. Thus, letting go of a long-held belief should be more difficult than integrating new information on an almost unknown subject. Importantly, even with similar levels of experience, individuals often differ in how confident they feel (Hoven et al., 2023; Navajas et al., 2017). While theoretical models and empirical evidence offer predictions about how confidence influences belief revision, the conditions under which beliefs become resistant to change remain poorly understood.

To address this gap, the present study focused on the role of initial expectations and confidence during the formation of novel ability beliefs and their subsequent revision. We used the Learning of Own Performance (LOOP) task, a validated experimental task that enables the capture of the formation processes underlying epistemologically novel beliefs, both self- and other-related, in the context of an estimation task (Czekalla et al., 2021; Müller-Pinzler et al., 2019, 2022). In this task, participants either estimated attributes of objects or observed another person estimating these attributes in different estimation categories (e.g., heights of buildings or weights of animals).

On each trial, participants received or observed manipulated performance feedback. By systematically linking estimation categories with predominantly “better-than-expected” or “worse-than-expected” feedback, participants were led to form novel ability beliefs about themselves (Müller-Pinzler et al., 2019). The condition in which participants observed feedback about another individual’s performance served as a control to disentangle belief formation processes specific to the self, as done in previous studies (Czekalla et al., 2021; Müller-Pinzler et al., 2019, 2022). We used cognitive estimation abilities as the target domain because such beliefs typically lack a strong pre-existing foundation and emerge from a relatively neutral starting point with low initial confidence. This design allows for the examination of belief formation mechanisms in a context less affected by prior learning histories than more personally salient domains such as academic (e.g., skills in mathematics) or athletic performance (Krach et al., 2024; Martinot et al., 2025).

To examine how initial expectations and confidence also impacted processes underlying belief revision, the LOOP task was employed in two consecutive sessions—a novel aspect of this study: The first session (belief formation; *T1*) was used to establish novel beliefs concerning one’s own or another individual’s cognitive estimation ability as done in previous work (Czekalla et al., 2021; Müller-Pinzler et al., 2019, 2022). In the second session (belief revision; *T2*), unbeknownst to the participants, feedback contingencies were reversed to probe how formerly established beliefs could be revised. Thus, the categories that predominantly elicited positive feedback at *T1* now at *T2* elicited predominantly negative feedback and vice versa. Due to the valuation of initial expectations and general motives of self-coherence (Elder et al., 2022; Müller-Pinzler et al., 2019), we hypothesized that belief formation at *T1* would be biased towards an individual’s initial belief. Furthermore, we hypothesized that confidence in beliefs should increase with learning during *T1*. Finally, we assumed that the more confident participants were in their beliefs after *T1*, the less inclined they would be to revise their beliefs based on new information during *T2*.

## METHODS

### Participants

A total of 102 participants (79 females) between the ages of 18 and 31 years (*M* = 22.49, *SD* = 2.82) took part in two studies with near identical setups (see Supplementary Note 1 for details). The two samples (*n*_*1*_ = 40, *n*_*2*_ = 62) were pooled for the analyses of this project. We excluded three individuals who did not believe the cover story of the task or did not complete the task attentively until the end (study 1: one exclusion, study 2: two exclusions), as indicated by self-reported tiredness and almost no variance in their expectation ratings. Thus, the final sample consisted of 99 participants, all were recruited from the University Campus of Lübeck, were fluent in German, and had either normal or corrected-to-normal vision. All participants provided written informed consent. The research received approval from the ethics committee at the University of Lübeck (reference number: AZ 21-217). It adhered to the ethical standards outlined by the American Psychological Association.

### The Learning of Own Performance Task (LOOP)

The LOOP task allows for the examination of how people gradually acquire knowledge about their own or another individual’s perceived cognitive abilities. The LOOP task has been previously introduced and validated in a series of studies describing the learning behavior and underlying neurocomputational processes involved when people arrive at novel ability beliefs (Czekalla et al., 2021; Müller-Pinzler et al., 2019, 2022). Unlike previous studies using the LOOP task, participants in this study completed the LOOP task over two sessions. In the first session (*T1*), novel beliefs were established, while in the second session (*T2*), these established beliefs were challenged using reversed feedback information.

### Belief Formation Using the LOOP Task

**Figure 1.**
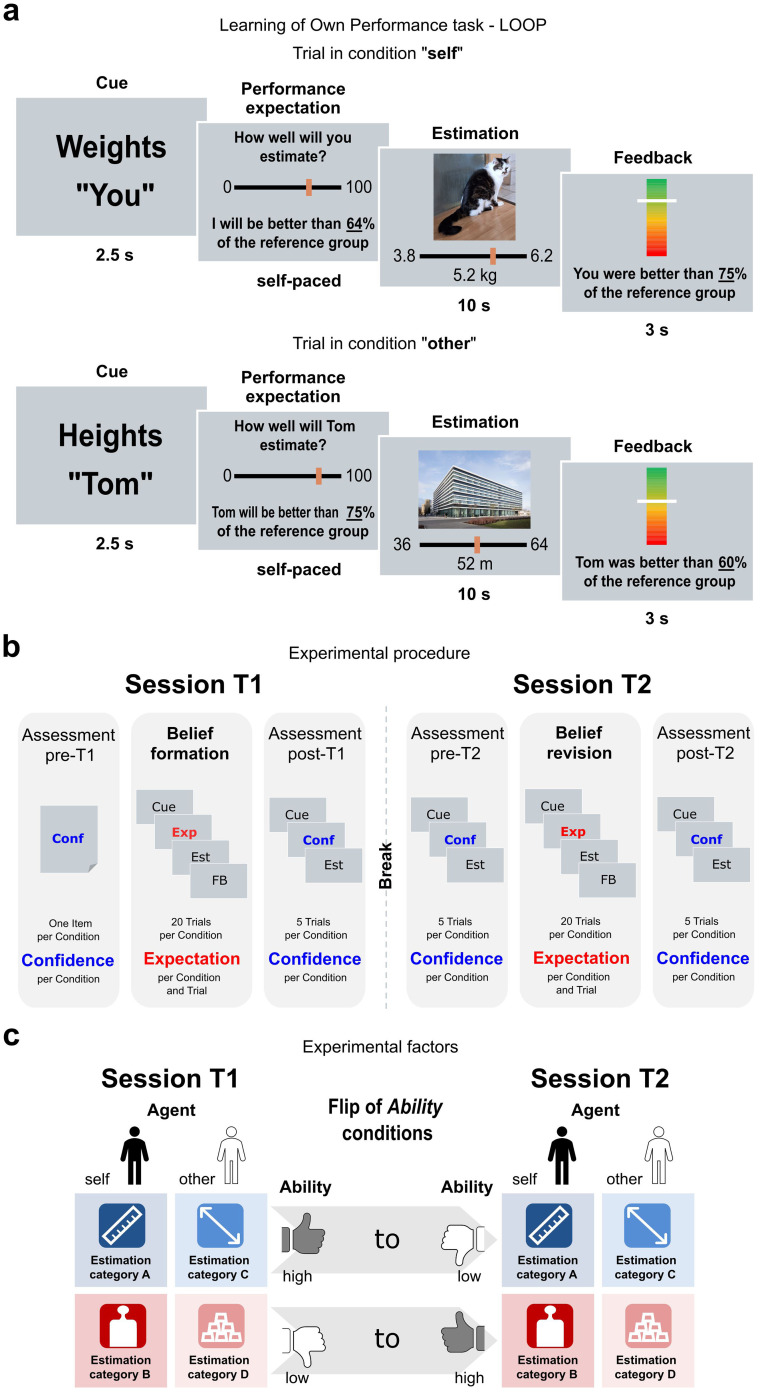
Learning of own performance (LOOP) task and study design. **(A)** Trial structure of the LOOP task. A cue prompted the estimation category (e.g., weights) and whether it was one’s own turn or the other person’s turn (e.g., you). Participants then provided their performance expectation rating and answered the actual estimation question, followed by feedback on their or the other person’s performance on that trial. **(B)** Experimental procedure. The study was divided into two sessions. At session *T1*, confidence was assessed in a pre-assessment. Subsequently, the LOOP task was conducted to encourage the formation of new beliefs within the respective categories, and performance expectations were measured for each trial and condition. A post-assessment was then carried out to determine confidence levels. This was achieved by presenting five new estimation questions for each condition, and by letting participants rate their confidence. Session *T2* started with an identical pre-assessment of confidence. To examine belief revision, the LOOP task was repeated, but now with reversed conditions of the factor *Ability*. Session *T2* ended with a final assessment of confidence. **(C)** Experimental conditions. The LOOP task was conducted on two consecutive sessions (factor *Session*: *T1* and *T2*) and additionally consisted of the factors *Ability* (*High Ability* vs. *Low Ability*; this determined whether participants were more likely to receive positive or negative feedback for the corresponding estimation category) and *Agent* (*Self* vs. *Other*). This resulted in four learning conditions that, for a given participant, were each bound to one of the four different estimation categories (heights, weights, quantities, and distances) allocated in randomized and counterbalanced order across participants. For session *T2*, the conditions of the factor *Ability* were reversed.

The experimental set-up is as follows: Participants were invited in pairs to take part in an experiment allegedly related to cognitive estimation. In cases where a second participant was not available, a trained confederate stepped in. Participants were informed that they would take turns (factor *Agent*) either performing the estimation task themselves (*Self*) or observing the other person perform it (*Other*). During each trial, participants received manipulated performance feedback in two distinct estimation categories, one associated with more positive feedback (70% positive PEs and 30% negative PEs) and the other with more negative feedback (30% positive PEs and 70% negative PEs; factor *Ability*: *High* vs. *Low Ability*). These conditions were assigned to the estimation categories pseudo-randomly (e.g., height of houses = *High Ability* condition and weight of animals = *Low Ability* condition, or vice versa), and the estimation categories were counterbalanced between *Ability* and *Agent* conditions (*Self* vs. *Other*). This resulted in four feedback conditions, each consisting of 20 trials (combinations of *Agent* and *Ability* conditions: *Self-High*, *Self-Low*, *Other-High*, *Other-Low*; Figure 1c). Performance feedback was provided after each estimation trial, indicating the participant’s or the other person’s current estimation accuracy compared to an alleged previously tested reference group of 350 university students. This feedback was conveyed in terms of percentiles (e.g., “You are better than 94% of the reference participants”). Instead of providing fixed feedback values, the feedback sequence was designed to evoke prediction errors (PEs) of specific magnitude and valence (positive vs. negative PEs). Providing feedback in this manner ensured that participants experienced comparable PEs across the study’s conditions, regardless of their initial expectations. More specifically, the feedback was based on each participant’s current ability belief, which was calculated for each trial as the average of their last five performance expectation ratings per category, plus a respective predefined modifier value (which was predominantly positive in the *High Ability* conditions (70%) and predominantly negative (30%) in the *Low Ability* conditions). Before participants provided their first performance expectation ratings (i.e., their initial expectation), this average was set to 50%. Overall, this ensured a relatively equal distribution of negative and positive PEs across *Self*- and *Other*-conditions (Supplementary Table 11).

At the start of each trial (see Figure 1a for the full sequence of a trial), a cue indicated the category (e.g., height) and the agent who was to perform the current trial (e.g., ‘you’ (*Self* condition) or ‘Tom’ (*Other* condition)). Participants were then asked to provide a performance expectation rating, either for their own expected performance (i.e., ‘you’ trials) or for the other person’s expected performance (i.e., ‘Tom’ trials). The participants did not interact directly with each other, i.e., they did not know how the other person evaluated their performance. This eliminated the interpersonal context that could have triggered moral or evaluative concerns, as has been shown to be relevant in other experimental settings (Siegel et al., 2018). The expected performance rating was provided at a self-determined pace on a scale that used the same percentiles as for the feedback. To motivate honest responses, participants were told that accurate expected performance ratings would be rewarded with up to 6 cents per trial. In other words, the closer their expectation rating matched their actual feedback percentile, the more money they would receive. Thus, reward incentives are linked to the accuracy of performance expectation rather than to supposed estimation performance (Czekalla et al., 2021; Müller-Pinzler et al., 2019, 2022). Following each performance expectation rating, participants had 10 seconds to answer the estimation question, with continuous response scales available for selecting plausible answers. Next, feedback was presented for 3 seconds (“You were better than 75% of the reference group.”).

### Belief Revision Using the LOOP Task

Participants completed the LOOP task again to assess belief revision up to 24 hours after the initial session *T1*. Participants at *T2* received estimation questions from the same categories that they had dealt with at *T1*. However, the feedback contingencies had now been reversed, and the participants were unaware of this fact (e.g., the estimation category ‘height of buildings’ switched from *High Ability* at *T1* to *Low Ability* at *T2* while the estimation category ‘weight of animals’ switched from *Low Ability* at *T1* to *High Ability* at *T2*; same for contingencies of condition *Other*). Consequently, this manipulation produced prediction errors that were identical in absolute magnitude across sessions but differed in valence. Both sessions of the task were executed using MATLAB Release 2019b and the Psychophysics Toolbox (Brainard, 1997; MATLAB, 2019).

### Assessment of Expectation and Confidence

The ratings for the first and last expectations per condition and session were used to assess *pre*- and *post*-*T1*/*T2* expectations. Additionally, participants rated their confidence in their own estimation abilities and in the abilities of the other participant. (Figure 1b). Pre- and post-task confidence ratings were assessed four times (factor *Timepoint*): At the beginning and the end of the belief formation session *T1* (*pre-T1* and *post-T1*), and at the beginning and the end of the belief revision session *T2* (*pre-T2* and *post-T2*). To measure confidence, participants were confronted with five new estimation stimuli from the same estimation categories as in the main task. They were asked to rate their confidence in their performance expectation rating, that is the confidence in their ability to predict their (or the other person’s) performance accurately (“How confident are you in this assessment?”). The scale ranged from zero (not certain at all) to 100 (very certain). At these stages, confidence was assessed without asking participants to perform the estimation task or providing feedback that could affect their confidence ratings. The mean values of these confidence ratings per combination of *Agent* × *Ability* and *Timepoint* were used for further analyses. The very first assessment of confidence (*pre-T1*) was conducted using a straightforward inquiry, omitting the presentation of particular stimuli. This approach was adopted to ascertain an initial assessment that remained uninfluenced by subsequent elements of the task.

### Questionnaires and Debriefing

Before the initiation of the experiment at *T1*, all participants were required to respond to a series of inquiries regarding demographic data. After completing the study at *T2*, participants underwent an interview that included assessments of their self-beliefs, were debriefed about the cover story, and were compensated for their time before departing. The entire procedure took approximately 3 hours—1.5 hours for each session.

### Statistical Analysis

#### Behavioral Analysis.

All analyses were done in R version 4.1.2 (R Core Team, 2021), unless stated otherwise. We initially conducted model-agnostic analyses on the participants’ performance expectation ratings and confidence ratings. To do so, we calculated several Linear mixed-effects models using the *lme4* package (Bates et al., 2015). For each dependent variable (expectation ratings at *T1*, expectation ratings at *T2*, confidence ratings) the maximal feasible random effects structures that still lead to model convergence were assessed using the *buildmer* package (Voeten, 2023). For expectation ratings, this stepwise procedure started from models with *buildmer* random effects structures for each session that included the *Ability* condition (*High Ability* vs. *Low Ability*) and *Agent* condition (*Self* vs. *Other*) as factorial variables and *Trial* (20 trials) as a continuous predictor and that treated the Intercept, *Ability* condition, *Agent* condition, and *Trial* as both fixed and random effects. For belief confidence, it started from a similar maximal model that included the *Ability* condition (*High Ability* vs. *Low Ability*) and the *Timepoint* (*pre-T1*, *post-T1*, *pre-T2*, *post-T2*) as factorial variables and as both fixed and random effects. After finding the maximal feasible random effects structures, these random effect structures were included in intercept-only models, and the respective fixed effects were added iteratively. The models were estimated using Maximum Likelihood (ML). The final models to be reported were then re-estimated using Restricted Maximum Likelihood (REML) as suggested by Meteyard and Davies (2020). To further explore specific effects or interactions of the final Linear mixed-effects models, where appropriate, pairwise comparisons of estimated marginal means were conducted using the package *emmeans* (Lenth, 2023). See Supplementary Notes 2 and 6 for further details and results.

### Computational Modeling

Following the completion of the model-agnostic analyses, the subsequent step involved the formal quantification of the dynamic changes in beliefs, with a particular focus on the performance expectation ratings. Therefore, we employed delta-rule update equations under the reinforcement learning framework (Rescorla & Wagner, 1972; Zhang et al., 2020). The basic equation used for these learning models is as follows (Equations 1 and 2; where ‘EXP’ represents performance expectation rating, ‘FB’ stands for feedback, ‘PE’ denotes prediction error, ‘*t*’ denotes the trial, and ‘*α*’ signifies the learning rate):

$${\text{EXP}}_{t+1}={\text{EXP}}_{t}+\alpha *{\text{PE}}_{t}$$ (1)

with

$${\text{PE}}_{t}={\text{FB}}_{t}-{\text{EXP}}_{t}$$ (2)

Within our model space (see Supplementary Figure 1 for a visual overview of the full model space), three primary models were explored, each with distinct assumptions regarding biased updating behaviors during belief formation. Building upon these primary models, several more complex variations were developed and tested, as explained in the following sections. While models varied in employed parameters, all of them included initial expectations about either one’s own or about the other participant’s performance (*SV* = starting values) as free model parameters.

#### Unity Model (M1).

The simplest learning model, the Unity Model, only differed in learning rates for *Self* vs. *Other*, thus assuming no valence-biases or biases that are linked to the factor *Ability*. This model assumes that there are no differences in the learning rates during belief formation compared to belief revision (*α*_*T1*_ = *α*_*T2*_).

#### Ability Model (M2).

The Ability Model, the second model, incorporated separate learning rates for each *Ability* condition (while still including different learning rates for *Self* vs. *Other*), indicating context-specific learning. This model assumes that belief formation and belief revision are primarily dependent on the experimental conditions in which learning takes place, specifically whether a category was associated with predominantly positive or negative feedback (factor *Ability*). Again, the same learning rates were applied to belief formation vs. belief revision (*α*_*T1*_ = *α*_*T2*_).

#### Valence Model (M3).

The Valence Model, the third model, introduced separate learning rates for positive PEs (*α*_PE+_) and negative PEs (*α*_PE−_) across both *Ability* conditions (while retaining different learning rates for *Self* vs. *Other*), suggesting that the valence (positive or negative) of the PE influenced belief formation and lead to potential bias. As with the first two models, the same learning rates were applied for *T1* and *T2* (*α*_*T1*_ = *α*_*T2*_), indicating no difference in belief formation vs. belief revision.

#### Models M4, M5, and M6—Belief Formation Differs From Belief Revision (*α*_*T1*_ ≠ *α*_*T2*_).

Additionally, we tested model variations of the three primary models: The first set of models described above (M1 to M3) assumed that individual learning rates did not vary between belief formation and belief revision (*α*_*T1*_ = *α*_*T2*_). The second set (M4 to M6) followed the logic of the introduced base models (M4 being a Unity Model, M5 an Ability Model, and M6 a Valence Model) but additionally assumed that individual learning rates varied between the two sessions (*α*_*T1*_ ≠ *α*_*T2*_).

#### Expectation Model (M8).

Building on the model variants that employed differing learning rates for *T1* and *T2* (*α*_*T1*_ ≠ *α*_*T2*_), two additional models were tested: The first one, the Expectation Model, was based on the Valence Model (M6). It separated learning rates across positive and negative PEs and additionally considered whether PEs of a specific valence were congruent with the majority of PEs within a condition or not. The rationale behind this model was that participants quickly learn to identify whether the outcomes within a condition are generally positive or negative. As a result, to some degree, participants may have gotten accustomed to PEs of a specific valence based on the estimation category in which they occur, similar to the degree to which participants learn to anticipate rewards in other tasks.

#### Extended Valence Model (M7)—The Winning Model.

**Figure 2.**
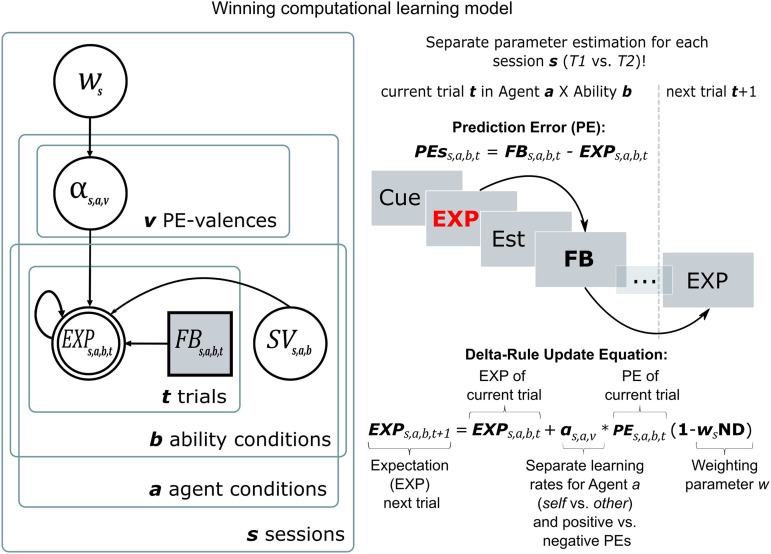
The winning computational model. The best-performing model assumed a difference in belief updating processes for sessions *T1* and *T2*. Performance expectations (EXP) per trial and combination of the experimental factors *Agent* (*Self* vs. *Other*) and *Ability* (*high* vs. *low*) were modeled through updates from prediction errors (PEs) for each participant. PEs resulted from the differences in trial-wise expectation ratings and the feedback (FB) received. Learning rates (α) were estimated separately for *Self* and *Others* and updates following positive (PE > 0) and negative (PE < 0) PEs (v = valence of PE). This resulted in four learning rates per session: *α*_self/PE+_, *α*_self/PE−_, *α*_other/PE+_, *α*_other/PE−_. This allowed a valence- and agent-specific characterization of the updating behavior across the initial belief formation phase of *T1* and the revision phase of *T2*. The winning model additionally contained a weighting parameter *w* per session, which reduced learning rates when receiving more extreme feedback (percentiles near the 0% or 100% mark). For this, each feedback percentile was mapped onto the relative probability density of the normal distribution (ND). Lastly, the model performed best when initial beliefs (SV = starting values; the initial expectation ratings) were estimated as free parameters for each combination of the *Agent* and *Ability* conditions, resulting in four more model parameters per session.

The Valence Model has consistently proven to be the most suitable in our previous studies (Czekalla et al., 2021; Müller-Pinzler et al., 2019). In the last publication, an extended version of the Valence Model was introduced (Müller-Pinzler et al., 2022) and therefore, was included in the current study as well. As an extension of the Valence Model, it distinguished learning rates by session (*α*_*T1*_ ≠ *α*_*T2*_). Additionally, a parameter *w* was introduced as a weighting factor to decrease learning rates when nearing the extremes of the feedback scale (percentiles close to 0% or 100%). The relative probability density of the normal distribution (ND) was associated with each feedback percentile value (see Supplementary Figure 2). The rationale behind this add-on was that more extreme feedbacks are perceived as less plausible and therefore discounted by participants during their belief updating. The participant- and session-specific weighting factor *w* was employed to reflect the degree to which the relative probability density influenced the reduction in learning rates for feedback far from the mean. Taken together, the following Extended Valence Model emerged (3, also see Figure 2), where ‘s’ represents the session (*T1* vs. *T2*), ‘a’ the *Agent* condition (*Self* vs. *Other*), ‘b’ the *Ability* condition (*High* vs. *Low*), and “v” the valence of the PE (PE+ vs. PE−):

$${\text{EXP}}_{s,a,b,t+1}={\text{EXP}}_{s,a,b,t}+\alpha_{s,a,v}*{\text{PE}}_{s,a,b,t}\left(1-w_{s}\text{ND}\right)$$ (3)

#### Mean Model (M9).

To assess whether participants’ performance expectation ratings could be better explained by PE learning rather than stable assumptions in each *Ability* condition, we also included a straightforward Mean Model with mean values for each task condition as a final control measure.

### Model Fitting

The RStan package (Stan Development Team, 2021) for the statistical computation language R (R Core Team, 2021) was used for model fitting with Markov Chain Monte Carlo (MCMC) sampling. For all participants, the models were fitted individually, and posterior parameter distributions were sampled. After 1,000 burn-in samples 2,400 samples were drawn (3,400 samples in total; thinned with a factor of three) using three MCMC chains. Convergence of the MCMC chains was checked by inspecting $\hat{R}$ values for each parameter. Effective sample sizes (*n*_*eff*_) of model parameters were checked to be mostly bigger than 1,500 (*n*_*eff*_ values are estimates of the effective number of independent draws from the posterior distribution of model parameters). To summarize posterior distributions of parameters, the mean was calculated, and thus, a single value per model parameter and participant was used for the following model-based analysis.

### Bayesian Model Selection (BMS)

**Figure 3.**
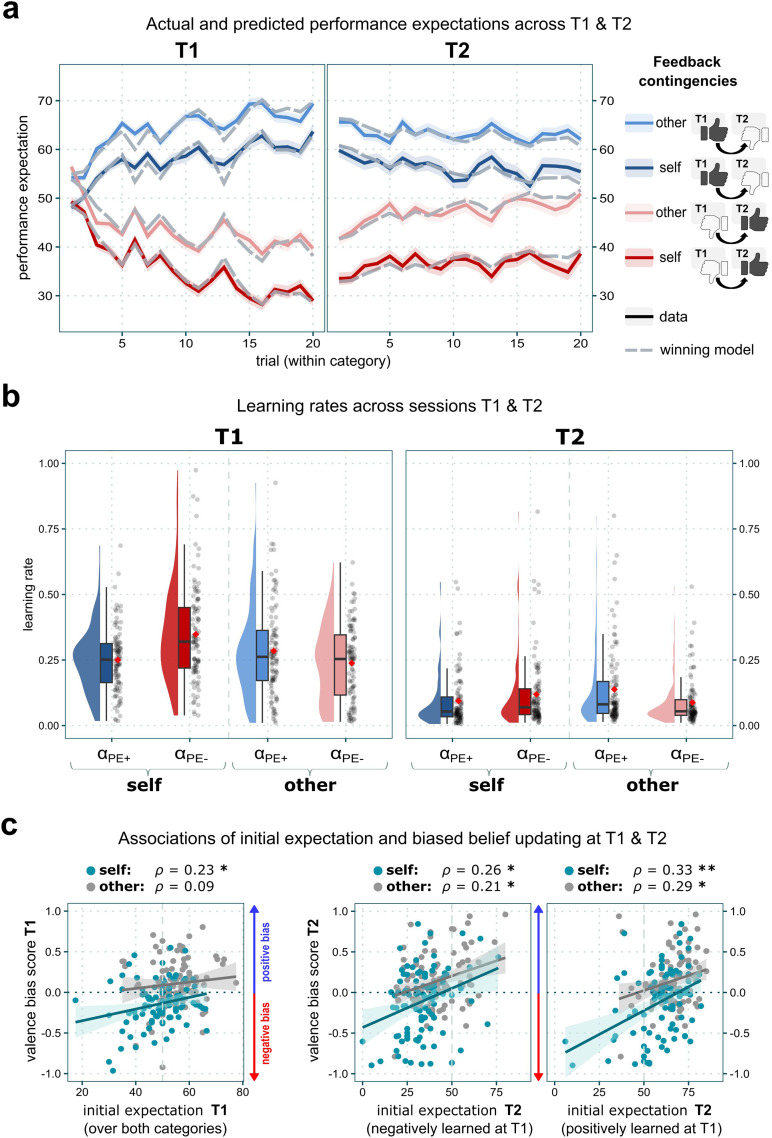
Performance expectation ratings across sessions *T1* and *T2* and learning parameters. **(A)** Actual mean performance expectation ratings (solid lines; shaded area: standard error) over time and mean ratings predicted by the winning model (dashed lines). During *T1*, participants adjusted their performance expectations according to the feedback given and formed distinct beliefs regarding their performance (*Self*) and the performance of another participant (*Other*) in four different domains. During *T2*, now confronted with feedback that violated the experience of the previous session, participants adjusted those beliefs to the new feedback to a lesser degree. **(B)** Self- and other-related Learning rates: Learning rates were significantly lower at *T2* than at *T1*. **(C)** Correlation plots of self- and other-related initial expectation and the valence bias score at *T1* and *T2*. For *T1*, the initial expectations were averaged over the two ability domains since these were still unaffected by the following experimental manipulations. *ρ* = Spearman’s rank correlation coefficient. Fitted lines only illustrate the overall trend of associations since rank correlations are used. *p < .05; **p < .001.

To determine the winning model, the pointwise out-of-sample prediction accuracy for all models of our model space was estimated separately for all participants using leave-one-out-cross-validation (LOO; in this case, one trial-observation was left out per participant; Acerbi et al., 2018; Vehtari et al., 2016). For this, Pareto smoothed importance sampling (PSIS) based on the log-likelihood calculated from the posterior parameter simulations was used as implemented by Vehtari et al. (2016). The sum of PSIS-LOO scores and the estimated shape parameters of the generalized Pareto distribution $\hat{k}$ were calculated. Only very few trials resulted in insufficient values for $\hat{k}$ and, therefore, in potentially unreliable PSIS-LOO scores (winning model *T1*: $\hat{k}$ > 0.7: 1.54%, winning model *T2*: $\hat{k}$ > 0.7: 0.39% (Vehtari et al., 2016); see Supplementary Table 1 for all PSIS-LOO scores and % $\hat{k}$ values > 0.7). To account for group heterogeneity in the model that best describes belief updating behavior, Bayesian Model Selection (BMS) on the PSIS-LOO Scores was performed (Rigoux et al., 2014) using the VBA (Variational Bayes approach) toolbox (Daunizeau et al., 2014) for Matlab. The protected exceedance probability (*pxp*) was provided for each model. The *pxp* value indicates how likely it is that a particular model explains the data with a higher probability than all other models in the considered model space. Bayesian omnibus risk (*BOR*) was calculated, which is defined as the probability of choosing a null hypothesis (in this case, a posterior probability that shows that model frequencies are all equal in a given model space) over an alternative hypothesis (Rigoux et al., 2014). In Supplementary Table 1 PSIS-LOO difference scores of the winning model in contrast to the other models of the model space are provided. These can be interpreted as a simple ‘fixed-effect’ model comparison (Acerbi et al., 2018; Vehtari et al., 2016). For our data, the comparisons of PSIS-LOO difference scores were generally comparable to the results of the BMS. To check if the data predicted by the winning model could capture the variance in performance expectation ratings of each participant, posterior predictive checks (Zhang & Gläscher, 2020; Zhang et al., 2020) were conducted by predicting the time course of expectation ratings for each participant and by comparing these predictions against the actual behavioral data for each session. Figure 3a visually confirms the model’s ability to capture the observed data for sessions *T1* and *T2*. In addition, we tested whether a model-agnostic analysis of the predicted data would capture the same effects as the analysis of the actual performance expectation ratings (see Supplementary Note 3). Since differences in belief updating for initial belief formation (*T1*) and belief revision (*T2*) seemed plausible, BMS was conducted separately for *T1* and *T2* to test whether different models fit best at different stages of belief updating.

### Statistical Analysis of Modeling and Behavioral Data

Learning rates of the winning model for each session were analyzed within-subject using Linear mixed-effects models in an approach identical to that used to analyze expectation and confidence ratings as described in ‘model-agnostic analyses’. We started from a maximal model that included the factors *Agent* (*Self* vs. *Other*), *PE-Valence* (*PE+* vs. *PE−*), and *Session* (*T1* vs. *T2*) as factorial variables and as both fixed and random effects (refer to Supplementary Note 4 for detailed results). The maximal feasible random-effects structure was assessed and included in an intercept-only model. Fixed effects were then added iteratively. Further, normalized valence bias scores (4) for each session were calculated to associate self- or other-related learning biases with (initial) expectations and confidence (Müller-Pinzler et al., 2019, 2022; Niv et al., 2012; Palminteri et al., 2017):

$$\text{Valence}\;\text{Bias}\;\text{Score}=\left(\alpha_{\text{PE}+}-\alpha_{\text{PE}-}\right)/\left(\alpha_{\text{PE}+}+\alpha_{\text{PE}-}\right)$$ (4)

A valence bias score greater than zero indicates greater belief updates after positive PEs compared to negative PEs, whereas a score below zero indicates greater updates after negative PEs compared to positive PEs. The valence bias scores for both sessions and conditions were then analyzed in the same way as learning rates, starting from a maximal model including the factors *Agent* (*Self* vs. *Other*) and *Session* (*T1* vs. *T2*). Spearman correlations were conducted between valence bias scores, individual learning rates, (initial) expectation ratings, and confidence. For correlation analyses, Benjamini-Hochberg false discovery rate (FDR) correction was applied to control for multiple testing. All statistical tests were performed two-sided.

## RESULTS

### Computational Modeling

The Extended Valence Model emerged as the winning model for both, belief formation phase (*T1*) and belief revision phase (*T2*): protected exceedance probability *pxp*_*T1*_ > .999; Bayesian Omnibus Risk *BOR*_*T1*_ < .001; estimated model frequency_*T1*_ = 81.98%; protected exceedance probability *pxp*_*T2*_ > .999; Bayesian Omnibus Risk *BOR*_*T2*_ < .001; estimated model frequency_*T2*_ = 68.86% (Supplementary Figure 3). Posterior Predictive Checks showed that the model adequately described the observed data (Figure 2a and Supplementary Note 3). The winning model (Figure 2) included separate learning rates (*α*) for positive and negative PEs for *Self* and *Other*, which allowed a *PE-valence*- and *Agent*-specific description of updating behavior. Additionally, it separated learning rates by the two task sessions, i.e., belief formation (*T1*) and belief revision (*T2*).

### Self-Specific Negativity Bias During Initial Belief Formation (T1)

During initial belief formation, participants displayed a self-specific negativity bias during learning, replicating earlier findings (Czekalla et al., 2021; Müller-Pinzler et al., 2019, 2022). That is, negative PEs led to higher updates as compared to positive PEs, as reflected by the learning rates. This effect was specific to the *Self*-condition (Figure 3b; interaction *Agent* × *PE-Valence* × *Session*: *β* = 0.07, 95% *CI* [0.01; 0.13], *t*_(490)_ = 2.15, *p* = .032; *α*_Self/PE+/T1_ − *α*_Self/PE−/T1_: *t*_(490)_ = −6.08, *p*_*Tukey*_ < .001). This negativity bias was also reflected in the negative valence bias score for *T1* (Supplementary Figure 4, *M* = −0.14, *SD* = 0.27). In comparison, the other-related valence bias score was positive and significantly higher (*M* = 0.11, *SD* = 0.29; Supplementary Figure 4) and showed an overall positively biased updating (main effect *Agent*: *β* = 0.25, 95% *CI* [0.17; 0.33], *t*_(98)_ = 5.96, *p* < .001). This indicates that individuals tend to learn more from negative feedback when forming beliefs about their performance.

### Lower Initial Expectations Are Linked to More Negative Self-Related Learning

Next, we examined how the initial expectations were related to biases in belief formation during *T1*, as indexed by the valence bias score. Lower initial expectations were associated with a more negative bias in self-belief formation (Figure 3c; initial expectation averaged across the two self-related conditions × valence bias score_Self/T1_: *ρ* = 0.23, 95% *CI* [0.03; 0.41], *p*_*FDR*_ = .035). This association was not found for the condition where participants were encouraged to form ability beliefs about the other person (*Other*: *ρ* = 0.09, 95% *CI* [−0.11; 0.29], *p*_*FDR*_ = .360). These results indicate that when it comes to how information is processed, there is a self-specific confirmation bias in the sense that feedback is processed in line with what participants already thought about themselves.

### Higher Learning was Associated With Higher Confidence During Belief Formation

**Figure 4.**
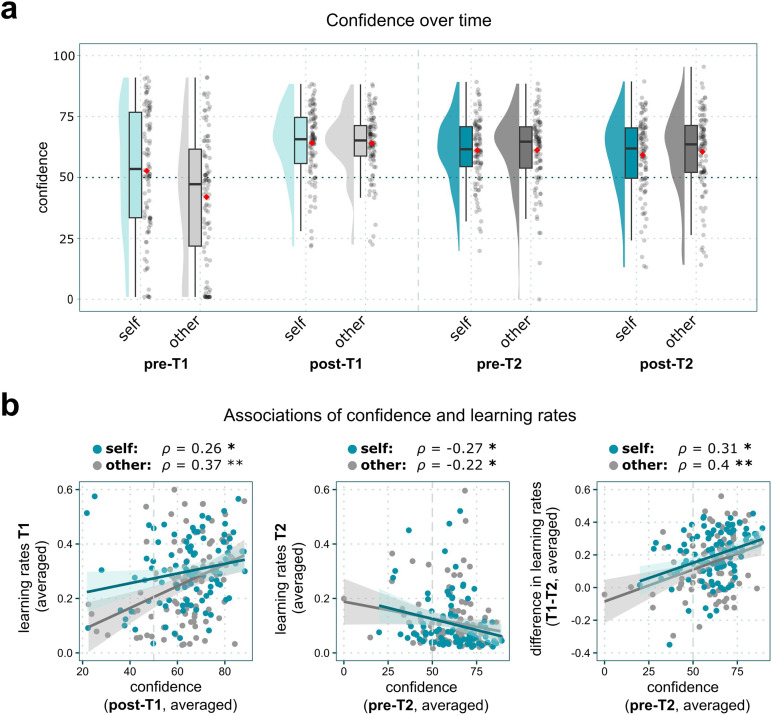
Confidence across sessions *T1* and *T2*. **(A)** Confidence over time: Confidence increased during *T1* but only slightly decreased over *T2* when feedback contradicted the experience of *T1*. Boxplots show median values (middle line), first and third quartiles (lower and upper lines), and mean values (red diamonds). Whiskers extend to data points within 1.5 times the interquartile range. **(B)** Correlation plots of confidence and learning rates (averaged over prediction error valence) across sessions. *ρ* = Spearman’s rank correlation coefficient. Fitted lines only illustrate the overall trend of associations since rank correlations are used. *p < .05; **p < .001.

Next, the participants’ confidence in their cognitive estimation abilities increased with learning and this increase was independent from the estimation category (Figure 4a; *post T1* vs. *pre T1*: *β* = 11.15, 95% *CI* [8.08; 14.23], *t*_(1401)_ = 7.12, *p* < .001, see Supplementary Note 6 for full analyses of confidence ratings), with a tendency towards higher ratings for self-related as compared to other-related learning (interaction *Agent* × *post T1* vs. *pre T1*: *β* = 10.98, 95% *CI* [6.64; 15.33], *t*_(1401)_ = 4.95, *p* < .001). The higher the self-related learning rates during session *T1* (averaged over *PE-Valence*), the more confident participants were about their belief at the end of *T1* (Figure 4b; *ρ* = 0.26, 95% *CI* [0.06; 0.43], *p*_*FDR*_ = .012). The same association could be observed for other-related confidence and average learning rate (*ρ* = 0.37, 95% *CI* [0.18; 0.53], *p*_*FDR*_ < .001). Thus, confidence in newly formed beliefs varied depending on how quickly new evidence was incorporated, irrespective of whether self- or other-related beliefs were formed (also see Supplementary Table 12).

### Diminished Learning During Belief Revision (T2)

To assess belief revision, we next examined the extent to which established beliefs from *T1* were updated when challenged at *T2*. At the onset of session *T2*, both expectation and confidence levels closely matched those observed at the end of *T1* (see Figure 3a, Figure 4a, and Supplementary Tables 2 and 3), indicating that participants maintained their beliefs across sessions. Upon exposure to reversed feedback contingencies in *T2*, learning rates in response to both positive and negative prediction errors were greatly reduced compared to the belief formation phase *T1* (main effect of *Session*: *β* = −0.16, 95% *CI* [−0.19, −0.12], *t*_(358.23)_ = −8.23, *p* < .001; Figure 3b).

Although no significant difference indicating an *Agent*-specific bias was found in learning rates for positive versus negative prediction errors at *T2* (*Agent* × *PE-Valence* × *Session*: *β* = 0.07, 95% *CI* [0.01; 0.13], *t*_(490)_ = 2.15, *p* = .032, *α*_*Self/PE+/T2*_ − *α*_*Self/PE−/T2*_: *t*_(490)_ = −1.56, *p*_*Tukey*_ = .405), valence bias scores continued to suggest a self-related negativity bias (*M* = −0.11, *SD* = 0.41; Supplementary Figure 4). In contrast, other-related scores during *T2* rather hinted towards a positive bias (*M* = 0.14, *SD* = 0.32; Supplementary Figure 4). However, there was no significant overall change in valence bias scores between sessions, regardless of *Agent* (main effect of *Session*: *β* = 0.03, 95% *CI* [−0.02, 0.08], *t*_(197)_ = 1.35, *p* = .178). This shows that although overall learning was greatly reduced during the revision phase—and thus the potential impact of a valence bias was limited—the tendency to exhibit a valence bias did not change significantly across sessions.

### Belief Revision was Biased in a Confirmatory Manner for Self- and Other-Related Learning

For belief revision, we again observed a consistent association between participants’ initial expectations about their performance at the beginning of *T2* and their valence bias scores (Figure 3c). Specifically, lower initial expectations predicted more negatively biased updating when learning about self-related abilities (former *Low Ability* category (negatively learned at *T1*)_Self_ × valence bias score_Self/T2_: *ρ* = 0.26, 95% *CI* [0.06; 0.44], *p*_*FDR*_ = .02; former *High Ability* category_Self_ × valence bias score_Self/T2_: *ρ* = 0.33, 95% *CI* [0.14; 0.5], *p*_*FDR*_ = .004). A similar pattern emerged for other-related belief revision (former *Low Ability* category_Other_ × valence bias score_Other/T2_: *ρ* = 0.21, 95% *CI* [0.01; 0.39], *p*_*FDR*_ = .046; former *High Ability* category_Other_ × valence bias score_Other/T2_: *ρ* = 0.29, 95% *CI* [0.1; 0.47], *p*_*FDR*_ = .010). These findings suggest that, despite an overall reduction in learning during *T2*, belief revision remained biased in a confirmatory direction: lower initial expectations were associated with more negative updates, for both self- and other-related beliefs. Taken together, these results indicate that individuals tended to maintain previously acquired beliefs, even when faced with contradictory feedback.

### Higher Confidence was Linked to Less Belief Revision

Despite receiving feedback that clearly contradicted the beliefs established at *T1*, confidence in beliefs about estimation abilities decreased only slightly at *T2* and remained significantly higher than at the beginning of *T1* (*pre-T2* vs. *pre T1*: *β* = 8.15, 95% *CI* [5.08; 11.22], *t*_(1401)_ = 5.20, *p* < .001; *post T2* vs. *pre T1*: *β* = 6.64, 95% *CI* [3.57; 9.71], *t*_(1401)_ = 4.23, *p* < .001). Significant interactions of *Agent × Timepoint* indicated that the initial difference between self- and other-related confidence decreased over time (*Agent ×* pre-T2 vs. *pre T1*: *β* = 10.91, 95% *CI* [6.57; 15.26], *t*_(1401)_ = 4.92, *p* < .001; *Agent × post T2* vs. *pre T1*: *β* = 11.94, 95% *CI* [7.60; 16.29], *t*_(1401)_ = 5.39, *p* < .001). Moreover, the results show that confidence was linked to belief revision (Figure 4b): The higher the participants’ initial confidence in their beliefs at *T2*, the less they revised their beliefs (averaged confidence_Self/pre-T2_ *×* averaged learning rates α_Self/T2_: *ρ* = −0.27, 95% *CI* [−0.44; −0.07], *p*_*FDR*_ = .012). These results indicate that confidence in one’s beliefs is linked to the extent to which those beliefs are maintained or revised. This relationship became even more apparent when the individual changes in learning rates from *T1* to *T2* were examined in relation to confidence at *T2*. Essentially, the more confident participants were before session *T2*, the greater their learning rates decreased during the revision session relative to *T1* (confidence_Self/pre-T2_ × averaged learning rates *α*_Self/T1-T2_: *ρ* = 0.31, 95% *CI* [0.12; 0.48], *p*_*FDR*_ = .003). Similar effects of confidence on learning rates during belief revision were found for the *Other*-condition (confidence_Other/pre-T2_ × averaged learning rates *α*_Other/T2_: *ρ* = −0.22, 95% *CI* [−0.4; −0.02], *p*_*FDR*_ = .031; confidence_Other/pre-T2_ × averaged learning rates *α*_Other/T1-T2_: *ρ* = 0.4, 95% *CI* [0.22; 0.56], *p*_*FDR*_ < .001).

## DISCUSSION

In the present study, we investigated the role of initial expectation and confidence during the formation of novel beliefs and, once established, during their revision. Belief formation was operationalized by allowing participants to gradually acquire new knowledge about either their own or another individual’s cognitive estimation abilities (Czekalla et al., 2021; Müller-Pinzler et al., 2019, 2022). Participants either received feedback on their own performance (self-belief formation) or observed feedback about another person’s performance (other-related belief formation). This feedback was systematically manipulated to be predominantly ‘better than expected’ or ‘worse than expected,’ prompting participants to update their beliefs accordingly. The rationale behind using cognitive estimation performance as a tool for describing the learning behavior underlying the development of a novel self-concept is that such beliefs originate from a rather neutral starting point, where individuals have minimal prior knowledge and low confidence in their judgements. This setup avoids the confounds associated with emotionally or motivationally charged belief domains such as mathematics, music, or athletic performance (Krach et al., 2024), thereby giving rise to fundamental mechanisms of belief formation and belief revision.

Our results demonstrate confirmatory updating tendencies at multiple stages. First, as hypothesized, the initial expectation of one’s estimation ability was associated with subsequent learning behavior: The lower the initial expectation, the more negatively participants processed the feedback and used this information to update their beliefs. At the same time, while the belief formation progressed, the confidence in these newly established beliefs increased. This effect was evident regardless of whether participants arrived at more positive or more negative beliefs and whether participants formed beliefs about their own abilities or those of another person. Further, higher learning rates were associated with higher confidence after belief formation. Second, once beliefs were established in the first session, they were relatively robust in the face of contradictory information in the second session. Furthermore, participants who were more confident in their beliefs before the second session were less likely to use the new, contradictory information to revise them. Together, these findings demonstrate that belief formation and belief revision—even in low-stakes, novel contexts—are guided by confirmatory processing. People tend to process information in a way that matches their prior expectations and stick to these beliefs even after such a short time and under controlled experimental conditions.

The present results are in line with evidence from research showing that individuals are motivated to gain consistency over their beliefs by processing information in a systematically biased manner, a phenomenon broadly described as confirmation bias (Hart et al., 2009; Kaanders et al., 2022; Palminteri, 2023; Palminteri & Lebreton, 2022; Talluri et al., 2018). For instance, individuals with strong academic self-beliefs may selectively attend to feedback that reinforces a positive self-view (McPartlan et al., 2021). Here, we examined the mechanisms underlying belief formation and how biased learning contributes to these processes. Extending previous work, we explored these dynamics in domains where participants had minimal prior knowledge and thus less initial confidence (i.e., beliefs about an agent’s ability to estimate weights, heights, quantities, or distances). Using somewhat artificial belief domains as a starting point enabled participants to form beliefs in either a more positive (e.g., “I think I am good at estimating the weight of animals”) or more negative direction (e.g., “I think I am bad at estimating the heights of buildings”) through a feedback loop paradigm. While we replicated our previous findings of a negativity bias during self-related belief formation (Czekalla et al., 2021; Müller-Pinzler et al., 2019, 2022), we extended these findings by showing that participants’ learning behavior matched their initial expectations. Participants with lower initial expectations about their estimation abilities exhibited more negatively biased updating, while those with higher initial expectations demonstrated relatively more positive (or less negative) learning. During the belief-formation process, this confirmatory pattern was specific to processing information related to oneself. Once beliefs were established after the first session, we found confirmatory learning patterns for both self- and other-related learning during the revision phase. These results suggest that previously observed learning asymmetries, such as the negativity bias found in previous studies (Czekalla et al., 2021; Müller-Pinzler et al., 2019, 2022), may, to some extent, be related to self-confirmatory updating tendencies that become apparent when initial expectations are minimal.

Furthermore, an increase in the confidence of the participants in their beliefs was observed as the task progressed. Consequently, the targeted feedback, which was more positive in one domain and more negative in the other, not only led to changes in expectations but also strengthened confidence in these beliefs as they developed. The change in confidence during belief formation had direct effects on processing contradictory feedback during the subsequent belief revision phase: once beliefs were experimentally established during the belief formation phase, participants were more confident and largely ignored disconfirming evidence during belief revision. Although participants still learned from the feedback during the belief revision phase, as indicated by learning rates greater than zero, these were significantly lower than during the belief formation phase. This attenuated updating with increasing confidence reflects another aspect of a confirmation bias, described as a lack of response to conflicting evidence (Klayman, 1995). This could indicate that individuals have implicitly prioritized maintaining a sense of self-consistency over optimizing belief accuracy (Bénabou & Tirole, 2016; Bromberg-Martin & Sharot, 2020) by strongly adhering to their previously formed self-beliefs. The finding of attenuated belief revision with increasing confidence is consistent with evidence from studies using probabilistic learning tasks, which showed that confidence guides updating behavior and learning from prediction errors (Meyniel, 2020; Meyniel & Dehaene, 2017). Notably, once established during the belief formation phase, confidence did not decrease significantly during the belief revision phase, although participants continuously received disconfirming information. In cases where new evidence has been accumulated and a novel belief is established, it could be reasoned that higher levels of confidence and maintenance of those levels are justified. In some cases, this may even be adaptive, protecting individuals against potentially erroneous updates. After all, while it is important to incorporate new information into our beliefs in order to navigate daily challenges (Bandura & Locke, 2003), it is also crucial to protect ourselves from updates resulting from random noise. Thus, a confidence-weighting mechanism could be a major aspect of adaptive learning (Meyniel, 2020). In addition, motives like promoting self-consistency and well-being also apply here. For example, it has been argued that (over-)confidence in one’s beliefs may have emerged from the motive of promoting self-consistency (Blanton et al., 2001). Moreover, being confident in one’s beliefs may in itself promote a sense of self-consistency and thus self-serving value in a way that is independent of objective belief accuracy (Sharot et al., 2023). This may also explain why, in the present study, participants underutilized the opportunity to revise their previously formed negative beliefs in light of more positive feedback during the revision phase. Since estimation abilities have little impact on everyday life, having high confidence in an (in)ability may be valuable enough.

These findings carry potential implications across educational, political, and clinical domains. In educational psychology, research has demonstrated a reciprocal relationship between self-beliefs and academic performance (Marsh & O’Mara, 2008; Marsh et al., 2006; Trautwein & Lüdtke, 2007). Within this framework, our results suggest that once students acquire a negative self-belief, e.g., “I am not good at math”, it may prove resistant to change, even when confronted with evidence to the contrary, particularly if such beliefs are held with high confidence. This aligns with recent findings by Martinot et al. (2025), who observed that gender stereotypes around mathematics emerge remarkably fast—within the first four months of schooling, leading girls to underperform despite comparable aptitude at entry. One possible explanation for this is that negative self-beliefs become entrenched at an early age, possibly due to gender stereotypes reinforced by the social environment. In the political domain, similar examples of rigid and hard-to-revise beliefs are observed (Light et al., 2022; Nyhan & Reifler, 2010). While political attitudes become self-relevant through context-dependent motivations, such as group identity (Tajfel et al., 1971; Tajfel & Turner, 1979) or personal stakes in political outcomes (Sharot et al., 2023), confidence again appears to play a critical role. For instance, during the COVID-19 pandemic, individuals who expressed the greatest dissent from scientific consensus often held the least accurate knowledge, yet were most confident in their views (Light et al., 2022). In clinical psychology and therapeutic contexts, our findings may inform interventions aimed at modifying maladaptive beliefs, particularly in disorders such as major depressive disorder, where negative schemas (Dozois & Beck, 2008; Schmidt & Joiner, 2004) and pessimistic belief updating are central features (Beck, 2002). Experimental work shows that symptom severity in depression is linked to diminished learning from positive feedback (Czekalla et al., 2024), alongside elevated confidence in one’s perceived lack of ability (Katyal et al., 2023). This pattern may reflect a form of ‘cognitive immunization’ (Kube et al., 2017, 2019; Rief & Joormann, 2019), wherein maladaptive beliefs persist due to insensitivity to corrective information. Consistent with this, research highlights that rigid negative expectations represent a core transdiagnostic mechanism across a range of mental disorders, contributing to the maintenance and generalization of maladaptive beliefs (Rief et al., 2015). Interventions that target metacognitive aspects, such as confidence in belief accuracy, may be essential for promoting belief revision (Wells, 2009). Without such an approach, patients may fail to incorporate even explicitly positive feedback (Czekalla et al., 2024). Enhancing the flexibility of belief updating by decoupling new learning experiences from rigid initial beliefs may thus be key to improving adaptive cognitive processes.

## CONCLUSION

This study demonstrates that once beliefs are formed, increasing confidence renders them more resistant to revision. Low initial expectations bias learning in a negative direction, and growing confidence solidifies these beliefs even in the face of contradictory evidence. These results extend the scope of confirmation bias to beliefs that are newly formed and experimentally induced. More broadly, the integration of information depends not only on what individuals believe, but how strongly they believe it. A deeper understanding of how expectation and confidence shape belief formation and revision processes may inform both theory and intervention across domains—from education and politics to mental health.

## ACKNOWLEDGMENTS

We want to thank Alica Steinert, Sophia von Krauss, Jovana Lehmann-Grube, Finn Moritz Borcherding, Laura Rosenbusch, and Clara Scheffel for their invaluable assistance with data collection.

## FUNDING INFORMATION

This research was funded by the German Research Foundation (Temporary Positions for Principal Investigators: MU 4373/1-1; MU 4373/1-3; Sachbeihilfe KR 3803/11-1; Sachbeihilfe KR 3803/41-1) and the Department of Medicine at the University of Lübeck, CS08-2023.

## AUTHOR CONTRIBUTIONS

A.S., N.C., L.M.P., S.D., and S.K. designed the research. A.S. and N.C. acquired the data. A.S. analyzed the data and prepared the manuscript. A.S., N.C., A.V.M., L.Z., D.S., C.W.K., S.D., F.L., F.M.P., and S.K. discussed the data analyses and interpretation of the results and reviewed and edited the manuscript.

## DATA AND CODE AVAILABILITY STATEMENT

The code and behavioral data for the statistical analyses are available at https://osf.io/8x4vp/ (DOI 10.17605/OSF.IO/8X4VP). *RStan* scripts to estimate the computational models are available from the corresponding author upon reasonable request.

## Supplementary Material


